# The National Medical Union

**Published:** 1914-02-21

**Authors:** 


### The National Medical Union.

THE Organisation Committee specially appointed to enrol members, link up non-panel associations, and spread a knowledge of the objects of the National Medical Union among isolated non-panel loyalists is now hard at work.

The heavy undertaking of this Committee would be infinitely lightened if each member of the Union would try to enlist new subscribers in his own district or communicate with friends in other counties where non-panel associations do not already exist, with the object of arousing the apathetic or stimulating the enthusiastic to still greater effort. The National Medical Union wants individual as well as collective co-operation and looks confidently to its members to work unceasingly for the cause.

THE FUTURE OF THE UNION.

As a fighting force it has already established itself firmly, and its early efforts in defence of the rights of non-panel practitioners have borne fruit and commanded respectful attention among opponents. What may happen in the political world to the Insurance Act in regard to amendment, repeal of the medical benefit section, abolition of compulsion, total repeal, or even a further attempt to extend its operations, no one, not even the Government, seems to be in a position to foretell. One thing is certain, which is that the Act as it stands is repulsive to more than half of the profession. Meanwhile it behoves all who have declined service to band together and support a federation which stands out by itself for the non-panel cause. There are none so busy as to be unable to find time to forward their subscription either to the treasurer of their local association (if there is one) or direct to the Central Offices, and there should be none so apathetic as to neglect at least to make themselves acquainted with what is being done in their interest.

ORGANISATION IN LONDON.

A meeting of the Organisation Sub-Committee for London took place at the offices of the Union on Saturday, February 14. Present: Dr. Oppenheimer (Hampstead) in the chair, Dr. Marsh (Westminster), Dr. Drake (Deptford), Dr. Handson (Deptford), Dr. Tilbury (Camberwell), Dr. Soden (Willesden), Dr. Shoyer (Islington and St. Pancras), Dr. Bott (Lambeth and Southwark), Dr. Greene (Wandsworth), Dr. Spurgin (Marylebone and St. John's Wood), and Dr. Carswell (Wandsworth). Apologies for absence were read from Dr. Maitland (Crystal Palace) and Dr. Hemmans (Lewisham), who were unavoidably prevented from being present.

The procedure to be adopted in carrying out a personal canvass and classification was arranged, and the various districts were mapped out. It was resolved to complete a first survey of the field within the next four weeks, after which the next meeting of the Sub-Committee will take place.

THE LIVERPOOL NON-PANEL UNION.

The annual meeting of the Liverpool Non-Panel Union was held on February 12 at the Medical Institution, Dr. O'Sullivan in the chair. Mr. Damer Harrisson was elected chairman of the Union in place of Dr. O'Sullivan, who was resigning on account of ill-health. Drs. O'Sullivan and Phillips Hunt were elected vice-chairmen, and the committee was re-elected with the addition of Dr. A. W. Montgomery to represent Liscard. Dr. George Parker was appointed treasurer and Dr. Heatherley was chosen to represent the Union on the Council of the Federation. Dr. Parker was instructed to send a list to each member of committee for the purpose of obtaining subscriptions. Drs. Brassey Brierley, Wheeler Hart, Skardon Prowse, and Sawers Scott were present at the meeting as visitors from Manchester.

